# Supplementary material for: iERM: An Interpretable Deep Learning System to Classify Epiretinal Membrane for Different Optical Coherence Tomography Devices: A Multi-Center Analysis
Source: J Clin Med. 2023 Jan 4;12(2):400. doi: 10.3390/jcm12020400 (PMC9862104; doi:10.3390/jcm12020400)
Supplement: Supplementary file 1 [file jcm-12-00400-s001.zip › jcm-2063210-supplementary.pdf]

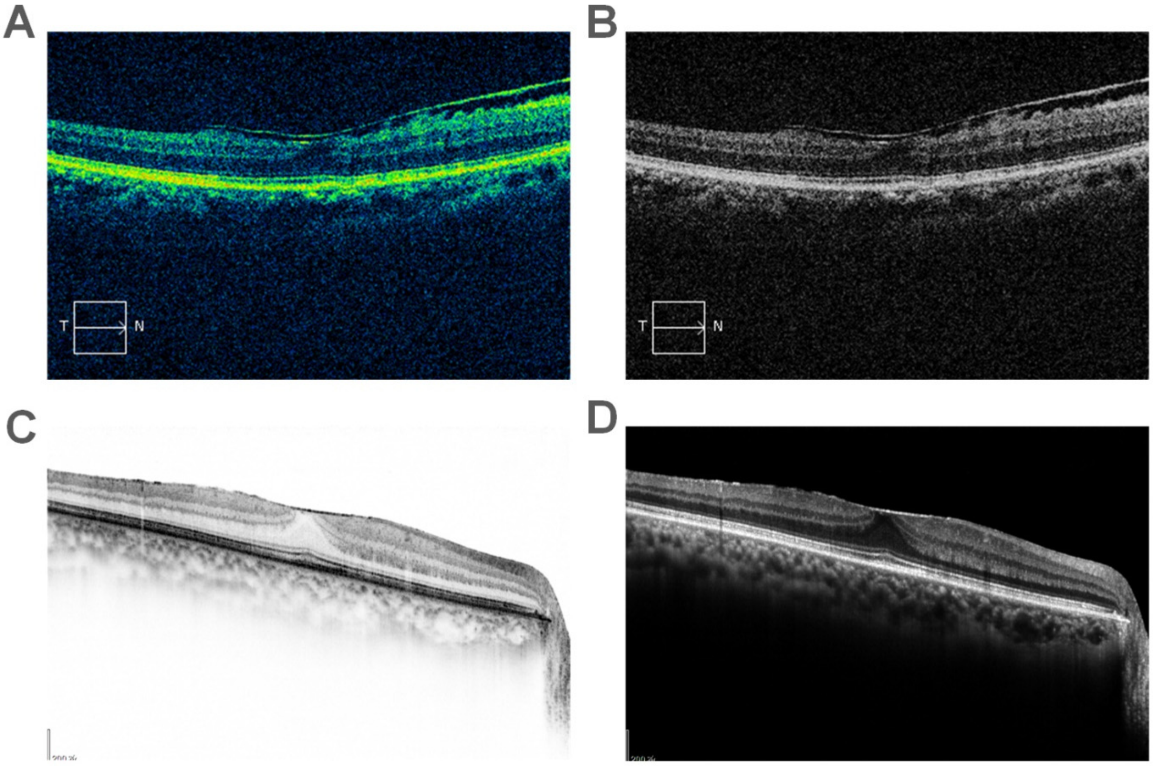


**Figure. S1.**

**The image mode conversion of the Zeiss image and Heidelberg 2 image.** (**A**) Zeiss OCT image (before image mode conversion). (**B**) Zeiss OCT image (after image mode conversion). (**C**) Heidelberg 2 OCT image (before image mode conversion). (**D**) Heidelberg 2 OCT image (after image mode conversion).

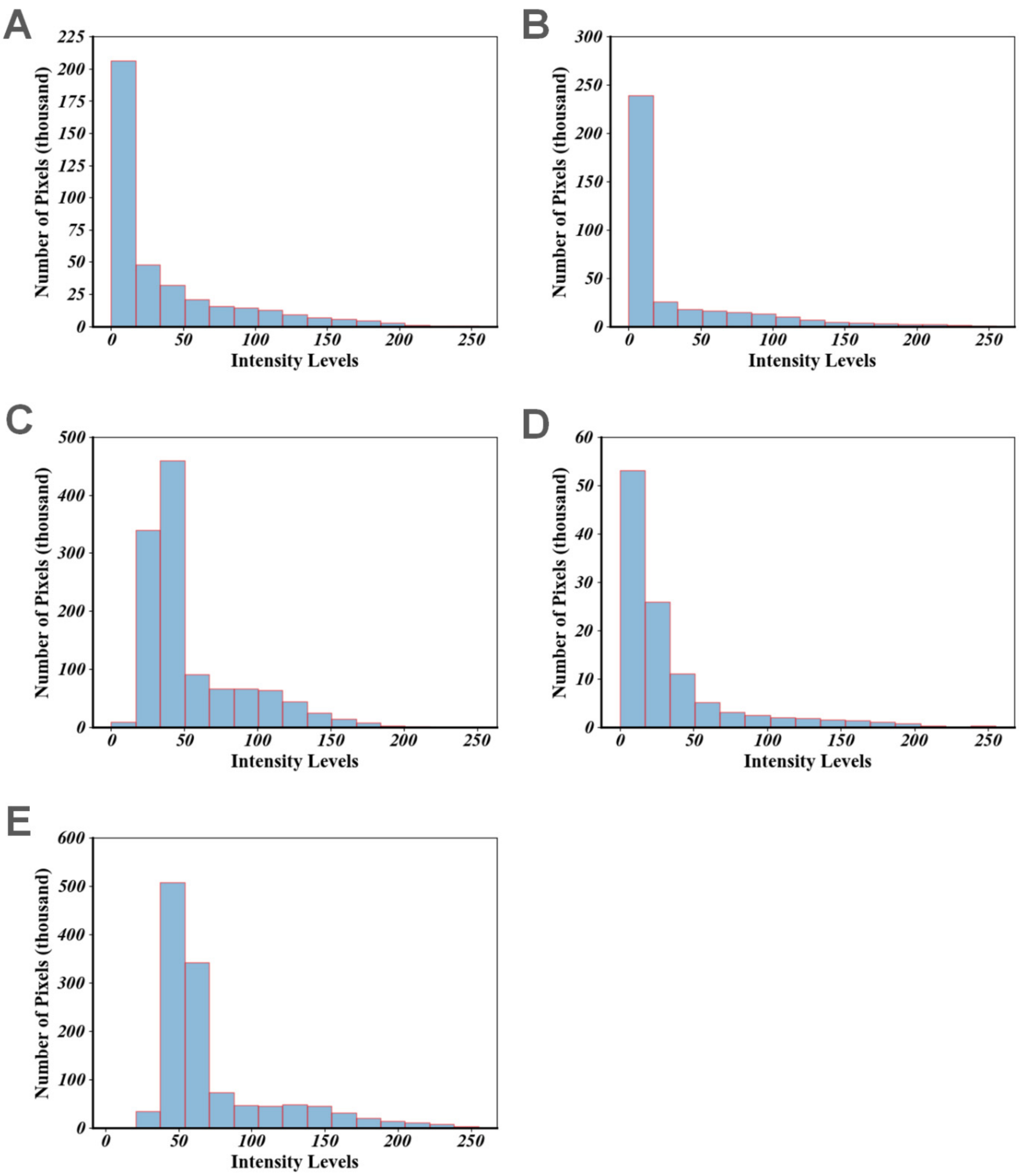


**Figure. S2.**

**The gray value distribution histograms of different devices.** (**A**) Heidelberg 1. (**B**) Heidelberg 2. (**C**) Crrius. (**D**) Zeiss. (**E**) Nidek.

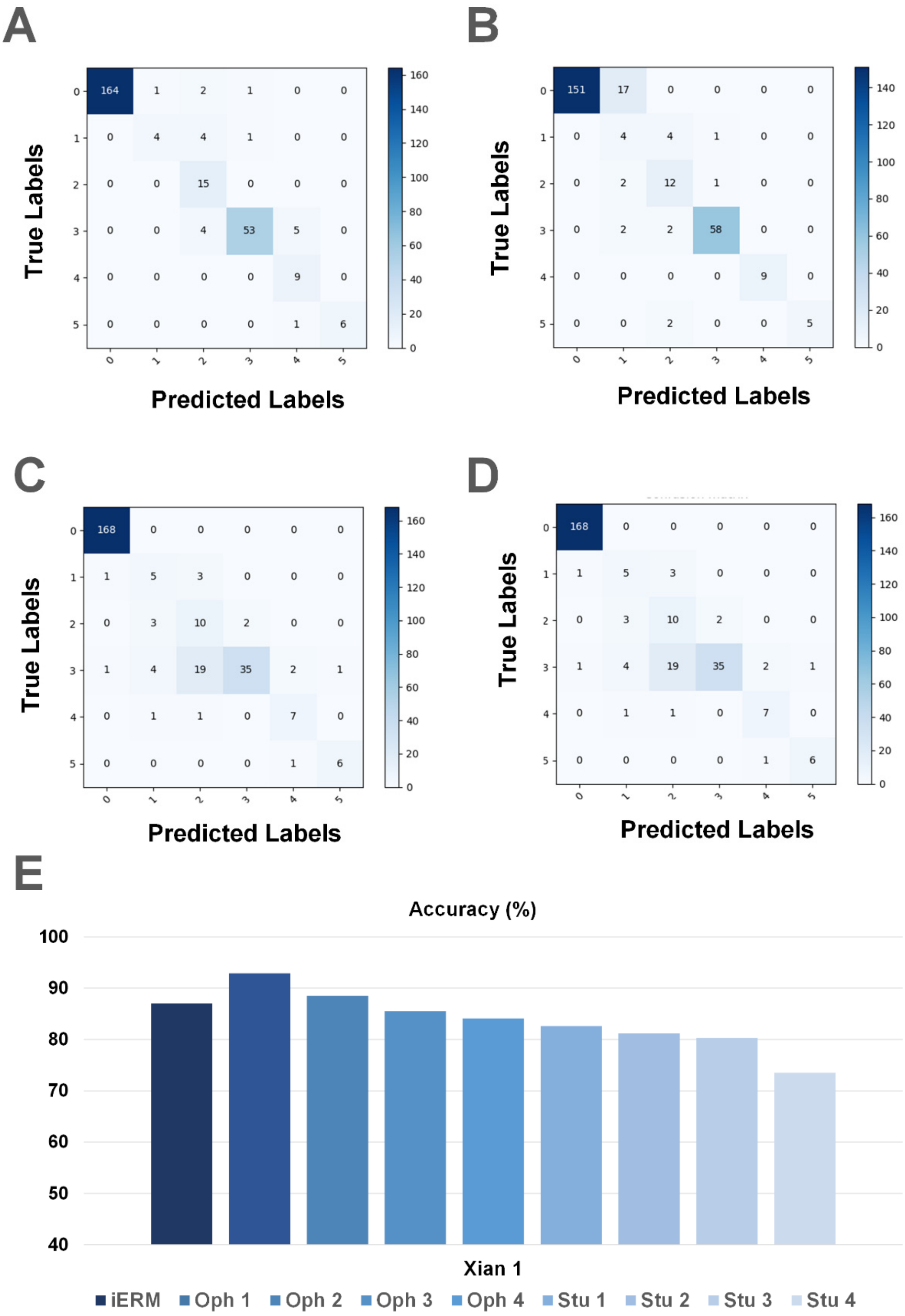


**Figure. S3.**

**Comparison of iERM with retinal ophthalmologists and medical students on the Xian 1 dataset.** (**A-D**) The confusion matrices of four retinal ophthalmologists. (**E**) the accuracy scores of iERM, four retinal ophthalmologists, and four medical students.

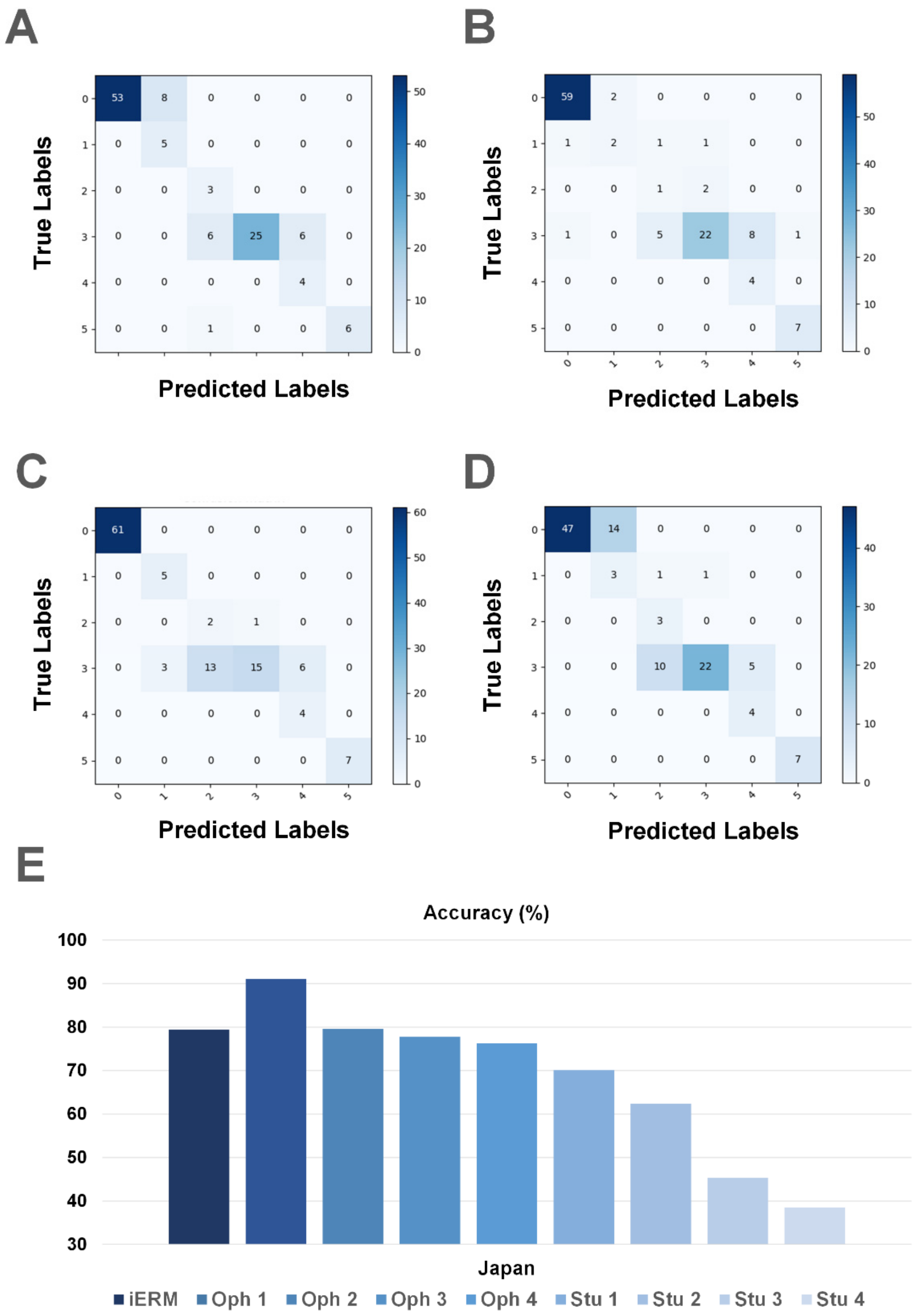


**Figure. S4.**

**Comparison of iERM with retinal ophthalmologists and medical students on the Japan dataset.** (**A-D**) The confusion matrices of four retinal ophthalmologists. (**E**) the accuracy scores of iERM, four retinal ophthalmologists, and four medical students.

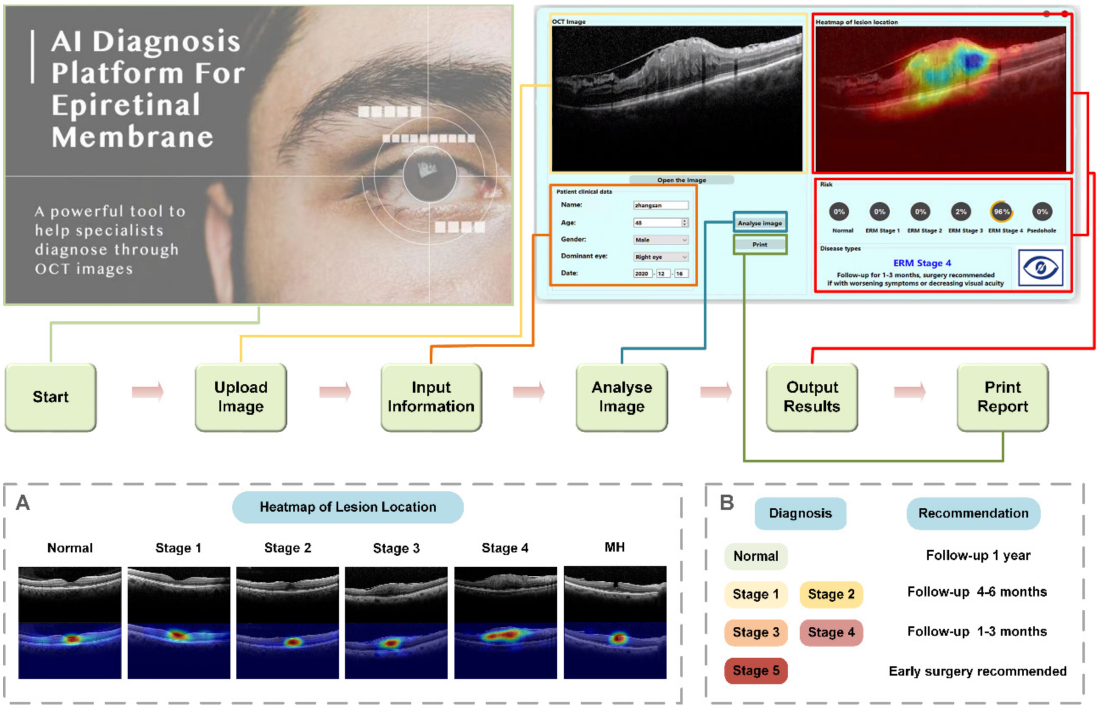


**Figure. S5.**

**The workflow of our user interface.** (**A**) The heat map of each stage. (**B**) The corresponding recommendation of each stage.

**Table. S1.**

**The specifications of images generated by each OCT device and the corresponding special processing.**

| OCT device | Image mode | Image size（pixel） | Special processing |
|---|---|---|---|
| Heidelberg 1 | Grayscale | 1344*882 | —— |
| Heidelberg 2 | Grayscale | 1609*763 | Gray value inversion |
| Crrius | Grayscale | 406*270 | —— |
| Zeiss | Pseudo-color | 768*496 | Image mode conversion |
| Nidek | Grayscale | 751*480 | —— |

**Table. S2.**

**The data distribution of training, validation, and test datasets for the classification task.**

| Dataset | Normal | Stage 1 | Stage 2 | Stage 3 | Stage 4 | Stage 5 | Total |
|---|---|---|---|---|---|---|---|
| Training | 1349 | 291 | 403 | 799 | 224 | 225 | 3291 |
| Validation | 192 | 41 | 57 | 114 | 31 | 32 | 467 |
| Internal test | 198 | 34 | 52 | 88 | 22 | 28 | 422 |
| External test Xian 1 | 168 | 9 | 15 | 62 | 9 | 7 | 270 |
| External test Japan | 61 | 5 | 3 | 37 | 4 | 7 | 117 |
| Total | 1968 | 380 | 530 | 1100 | 290 | 299 | 4567 |

**Table. S3.**

**The dataset-based distribution of image data usage for the classification task.**

| Dataset | OCT device | Total number | Training | Validation | Internal test | External test |
|---|---|---|---|---|---|---|
| ZJU | Heidelberg | 3557 | 2734 | 401 | 422 | 0 |
| Xian 1 | Heidelberg | 270 | 0 | 0 | 0 | 270 |
| Ningbo | Heidelberg | 84 | 74 | 10 | 0 | 0 |
| Jinhua | Heidelberg | 126 | 110 | 16 | 0 | 0 |
| Dali | Heidelberg | 121 | 106 | 15 | 0 | 0 |
| Anhui | Heidelberg | 19 | 17 | 2 | 0 | 0 |
| Japan | Heidelberg | 117 | 0 | 0 | 0 | 117 |
| Singapore 1 | Heidelberg | 71 | 62 | 9 | 0 | 0 |
| Taizhou | Nedik | 22 | 20 | 2 | 0 | 0 |
| Xian 2 | Zeiss | 109 | 89 | 13 | 0 | 0 |
| Singapore 2 | Cirrus | 78 | 69 | 9 | 0 | 0 |

**Table. S4.**

**The results of the segmentation task. Dice: the Dice coefficient. IoU: mean intersection over union.**

| | **Layer 1** | **Layer 2** | **Layer 3** | **Layer 4** | **Layer 5** | **Mean** |
|---|---|---|---|---|---|---|
| Dice | 0.997 | 0.955 | 0.896 | 0.962 | 0.958 | 0.953 |
| Precision | 0.997 | 0.948 | 0.889 | 0.965 | 0.962 | 0.952 |
| Recall | 0.997 | 0.963 | 0.906 | 0.961 | 0.955 | 0.956 |
| Mean IoU | 0.994 | 0.915 | 0.815 | 0.929 | 0.920 | 0.915 |
| ASD (mm) | 0.014 | 0.119 | 0.308 | 0.119 | 0.915 | 0.130 |

**Table. S5.**

**The results of three traditional classification models in the internal dataset.**

| Internal | Normal | Stage 1 | Stage 2 | Stage 3 | Stage 4 | Stage 5 |
|---|---|---|---|---|---|---|
| **ResNet-34** | | | | | | |
| Precision (%) | 96.5 | 69.4 | 61.1 | 65.1 | 88.2 | 80.0 |
| Recall (%) | 98.5 | 73.5 | 42.3 | 78.4 | 68.2 | 71.4 |
| F1-score (%) | 97.5 | 71.4 | 50.0 | 71.1 | 76.9 | 75.5 |
| AUC (%) | 99.8 | 92.4 | 90.9 | 90.4 | 97.0 | 98.3 |
| Accuracy (%) | | | 81.9 | | | |
| **Inception-v3** | | | | | | |
| Precision (%) | 97.3 | 50.0 | 57.3 | 67.1 | 60.0 | 73.3 |
| Recall (%) | 90.4 | 67.6 | 83.7 | 62.5 | 13.6 | 78.6 |
| F1-score (%) | 93.7 | 90.3 | 67.7 | 64.7 | 22.2 | 75.9 |
| AUC (%) | 99.2 | 64.6 | 92.3 | 92.4 | 97.8 | 95.0 |
| Accuracy (%) | | | 77.0 | | | |
| **EfficientNet-b0** | | | | | | |
| Precision (%) | 94.7 | 93.3 | 58.5 | 62.8 | 68.2 | 65.9 |
| Recall (%) | 100.0 | 41.2 | 46.2 | 67.0 | 68.2 | 96.4 |
| F1-score (%) | 97.3 | 57.1 | 51.6 | 64.8 | 68.2 | 78.3 |
| AUC (%) | 99.7 | 93.4 | 90.8 | 91.6 | 97.9 | 98.9 |
| Accuracy (%) | | | 79.8 | | | |

**Table. S6.**

**The results of iERM in the internal dataset compared with those of the traditional classification model.**

| Internal | Normal | Stage 1 | Stage 2 | Stage 3 | Stage 4 | Stage 5 |
|---|---|---|---|---|---|---|
| **without segmentation** | | | | | | |
| Precision (%) | 96.5 | 69.4 | 61.1 | 65.1 | 88.2 | 80.0 |
| Recall (%) | 98.5 | 73.5 | 42.3 | 78.4 | 68.2 | 71.4 |
| F1-score (%) | 97.5 | 71.4 | 50.0 | 71.1 | 76.9 | 75.5 |
| AUC (%) | 99.8 | 92.4 | 90.9 | 90.4 | 97.0 | 98.3 |
| Accuracy (%) | | | 81.9 | | | |
| **with segmentation** | | | | | | |
| Precision (%) | 98.0 | 75.0 | 62.0 | 69.7 | 64.0 | 80.0 |
| Recall (%) | 99.5 | 70.6 | 59.6 | 70.5 | 72.7 | 71.4 |
| F1-score (%) | 98.7 | 72.7 | 60.8 | 70.1 | 68.1 | 75.5 |
| AUC (%) | 99.9 | 90.4 | 92.5 | 92.8 | 97.4 | 99.0 |
| Accuracy (%) | | | 82.9 | | | |

**Equations of evaluation index:**

**1. For the segmentation task:**

In the following equations, X is the segmentation prediction and Y is the segmentation ground truth.

Dice coefficient (Dice):

$$\mathrm{Dice(X,Y)} = \frac{2|X \cap Y|}{|X| + |Y|}$$

Precision:

$$\mathrm{Precision(X,Y)} = \frac{|X \cap Y|}{|X|}$$

Recall:

$$\mathrm{Recall(X,Y)} = \frac{|X \cap Y|}{|Y|}$$

Intersection over union (IoU):

$$\mathrm{IoU(X,Y)} = \frac{|X \cap Y|}{|X \cup Y|}$$

Average surface distance (ASD):

$$\mathrm{ASD}(\mathrm{X}, \mathrm{Y}) = \frac{\sum_{x \in \mathrm{X}} \min_{y \in \mathrm{Y}} d(x, y)}{|\mathrm{X}|}$$

Where d(x, y) is the distance between the two points x, y.

**2. For the classification task:**

In the following equations

True Positive(TP)： Number of positive class predicted as positive class

True Negative(TN)： Number of negative class predicted as negative class

False Positive(FP)： Number of positive class predicted as negative class

False Negative(FN)： Number of negative class predicted as positive class

Precision:

$$\text{Precision} = \frac{TP}{TP + FP}$$

Recall:

$$\text{Recall} = \frac{TP}{TP + FN}$$

F1-score:

$$\text{F1-score} = \frac{2 * \text{Pre}cision * Re\,c\,all}{\text{Pre}cision + Re\,c\,all}$$

The area under the curve (AUC) of the receiver operating characteristic (ROC) curve:

$$\text{AUC} = \frac{\sum_{i \in positiveClass} rank_i - \frac{M(1+M)}{2}}{M \times N}$$

Where M is the number of positive class samples and N is the number of negative class samples.

Total accuracy:

$$\mathrm{ACC} = \frac{TP + TN}{TP + TN + FP + FN}$$
